# Unmasking of intracranial metastatic melanoma during ipilimumab/nivolumab therapy: case report and literature review

**DOI:** 10.1186/s12885-018-4470-y

**Published:** 2018-05-09

**Authors:** Marin A. McDonald, Parag Sanghvi, Julie Bykowski, Gregory A. Daniels

**Affiliations:** 1 UC San Diego Health Department of Radiology, 200 W. Arbor Drive MC 0834, San Diego, CA 92103-0834 USA; 2 UC San Diego Health Department of Radiation Medicine and Applied Sciences, 9500 Gilman Drive, La Jolla, CA 92093 USA; 3 Department of Medicine, UC San Diego Health Moores Cancer Center, 3855 Health Sciences Drive, La Jolla, San Diego, CA 92093 USA

**Keywords:** Immunotherapy, Checkpoint blockade, Metastatic melanoma, Intracranial metastases

## Abstract

**Background:**

While data from several studies over the last decade has demonstrated that introduction of immunologic checkpoint blockage therapy with anti-CTLA-4/PD-1 drugs leads to improved survival in metastatic melanoma patients, relatively little is known about brain-specific therapeutic response and adverse events in the context of immunotherapeutic treatment of intracranial disease. Here we report two independent cases of new intracranial metastases presenting after initiation of combined checkpoint blockade Ipilimumab and Nivolumab for recurrent metastatic melanoma in the context of positive systemic disease response.

**Case presentation:**

Case #1: A 43-year-old Caucasian male with Stage III melanoma of the left knee had subsequent nodal, hepatic and osseous metastases and was started on ipilimumab/nivolumab. He developed an intractable headache one week later. MRI revealed new enhancing and hemorrhagic brain metastases. After 6 weeks of immunotherapy, there was interval hemorrhage of a dominant intracranial lesion but substantial improvement in systemic metastatic disease. Durable, near complete intracranial and systemic response was achieved after completion of both induction and maintenance immunotherapy.

Case #2: A 58-year old Caucasian woman with stage II melanoma of the right index finger developed cutaneous, pulmonary and hepatic metastases within 4 months of adjuvant radiation. Although combined checkpoint blockade resulted in improvement in both cutaneous and systemic disease, brain MR performed for eye discomfort demonstrated new enhancing and hemorrhagic brain metastases. Serial MR imaging five months later revealed only a solitary focus of brain enhancement with continued improved systemic disease.

**Conclusions:**

These cases raise the question of whether the initial immune activation and modulation of the blood brain barrier by Ipilimumab/Nivolumab somehow “unmasks” previously clinically silent metastatic disease, rather than representing new or progressive metastatic disease. An overview of currently available literature discussing the role of immune checkpoint blockade in the treatment of intracranial metastatic melanoma will be provided, as well as discussion highlighting the need for future work elucidating the response of brain metastases to anti-CTLA/PD-1 drugs and documentation of brain-specific adverse events.

## Background

Nearly half of patients with advanced melanoma develop intracranial metastasis over the course of their disease [1–4]. Before the availability of anti-CTLA-4/PD-1 drugs, the diagnosis of brain metastases portended a dismal prognosis, with median overall survival of approximately 6 months [1]. Data from several recent phase 3 studies demonstrated that the introduction of immunologic checkpoint blockade therapy leads to improved survival of metastatic melanoma patients, with medial survival ranging between 10 and 25 months [5–8]. Accordingly, much is known about the kinetics of response in patients with extracranial disease, which can encompass early response, delayed response, pseudo- or frank progression. However, there is a relative paucity of clinical data for intracranial disease response to immunotherapy, as these patients are often under-represented or excluded from the majority of clinical trials [2]. While early data suggests single agent checkpoint blockade has similar safety and activity within the CNS [9–12], little is known about the CNS-specific pattern of response and immune-related toxicities. In this report we describe two cases of advanced melanoma treated with ipilimumab and nivolumab (ipi/nivo) checkpoint blockade which developed intracranial enhancing and hemorrhagic lesions in the context of positive systemic therapy response.

## Case presentation

### Case #1

A 43 year old Caucasian male had a wide local excision of a changing pigmented lesion on his left knee in 2009 (Stage III *BRAF V600*) with biopsy proven local recurrence and multiple left inguinal lymph node metastases developing within the ensuing 2 years. Brain MRI performed at the time was negative for intracranial metastatic disease. Over the next 3 years, he underwent adjuvant chemotherapy but progressed with recurrent left iliac and pelvic nodal involvement, hepatic and osseous metastases. In November 2014, he received a single cycle of ipi/nivo (295 mg/90 mg), complicated by transaminitis and a subcapsular hepatic hemorrhage. Subsequent treatment included dabrafenib (150 mg twice daily), tramentinib (2 mg daily) for two months followed by standard high dose IL-2. Follow-up brain MRI in March 2015 showed no evidence of intracranial involvement, although continued progression with right pelvic node and multiple soft tissue lesions became evident in December 2015.

**Fig. 1:**
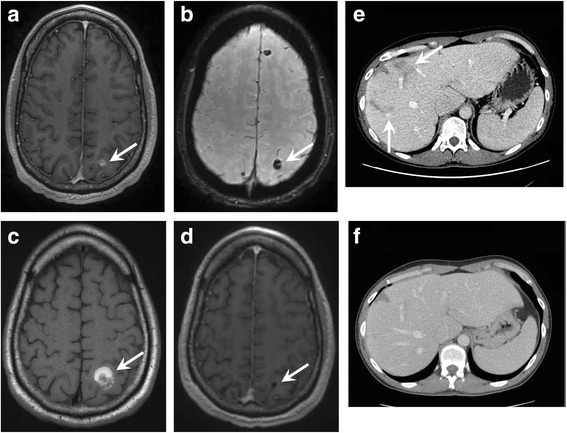
Axial post-contrast FSPGR obtained 1 week after the start of ipi/nivo due to new headaches shows a new 6 mm enhancing (**a**, arrow) and hemorrhagic left parietal lesion (**b**, arrow), one of 9 new lesions. 6 week follow-up MRI during ipi/nivo therapy revealed interval hemorrhage of the left parietal lesion (**c**, arrow) and decreased size of the additional previously enhancing lesions. Axial images from concurrent abdominal CT examinations demonstrate systemic disease response with decreased size and number of hepatic lesions after 6 weeks of ipi/nivo therapy (arrows in **e** compared to **f**). After 6 months of therapy, MRI confirmed contraction of the hemorrhagic cavity of the left parietal metastasis (**d**, arrow) and no new/residual enhancing lesions

The patient was re-started on ipi/nivo (300 mg/90 mg every 2 weeks) in March 2016 and within a week developed new headaches. Brain MRI at that time revealed multiple enhancing and hemorrhagic lesions (Fig. 1a, b) which were new compared to the MRI performed one year prior. The patient did not receive brain radiotherapy at any time during the course of his treatment. Instead, ipi/nivo therapy was continued and on repeat MRI 6 weeks later many of the smaller parenchymal lesions were no longer enhancing. Although there was evidence of interval hemorrhage of the dominant, left parietal lesion (Fig. 1c), his prior symptoms had resolved and he had no focal neurologic deficits. Concurrent CT imaging revealed a substantial reduction in the size of metastatic lesions involving the liver, greater omentum and left pelvic wall (Fig. 1e, f). He was continued on ipi/nivo for four months (280 mg/90 mg every 2 weeks) and surveillance MRI performed 6 months after the initiation of this round of immunotherapy showed further contraction of prior parenchymal hemorrhages and no residual or new enhancing metastases (Fig. 1d). The patient is currently continuing on maintenance nivolumab therapy (240 mg every 2 weeks) with no evidence of new or progressive intracranial disease at the time of this publication (12 months after the initiation of immunotherapy).

### Case #2

A 58-year old Caucasian woman was treated with wide local excision of melanoma of the right index finger (T3B, *BRAF V600E*) and had a negative right axillary sentinel node dissection in 2014. Within 4 months of completing 15 treatments of adjuvant radiation to the distal finger she developed a right forearm lesion, with additional bilateral lung metastases and a single hepatic lesion two months later. She started pembrolizumab, dabrafenib (150 mg twice daily) and tramentinib (2 mg daily) at an outside institution, with significant decrease in the size of the intrapulmonary and intrahepatic lesions but mildly progressed intra-abdominal nodal involvement. After significant progression of multiple skin lesions in the ensuing three months, she started a trial of LGX818/MEK162, however surveillance revealed multiple new soft tissue nodules within the mediastinum, abdomen, bilateral breasts and right axilla. While she underwent radiation to the right arm in March 2016 (24 Gray in 3 fractions), MRI of the right upper extremity in April 2016 revealed interval growth of the primary lesion and multiple associated subcutaneous nodules. Brain MRI at that time revealed no intracranial metastases.

**Fig. 2:**
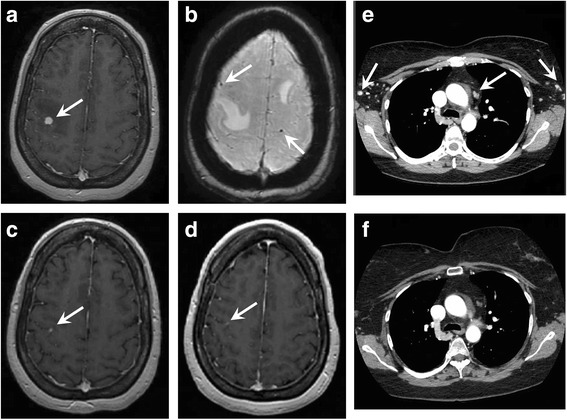
Axial post-contrast FSPGR brain MRI was performed 3 months after the initiation of ipi/nivo in the setting of new neurologic symptoms, revealing 6 new enhancing parenchymal lesions, the largest in the right parietal lobe (**a**, arrow). Multiple additional foci of chronic hemorrhage without associated visible enhancement are demonstrated on susceptibility-weighted imaging (**b**, arrow). Concurrent thoracic CT revealed interval decrease in size and contrast enhancement of several axillary and mediastinal lymph nodes (compare arrows in **e** with **f**). Post-contrast MRI performed one month later showed marked decrease in the size of the right parietal lesion (**c**, arrow), and resolution of all of the previously enhancing lesions. The patient was tapered off steroids without significant change in imaging after four months (**d**, arrow)

In April 2016, ipi/nivo was initiated (250 mg/90 mg every 2 weeks) with resulting decreased size of multiple clinically visible cutaneous nodules involving the right upper extremity over the course of 4 cycles of ipi/nivo. She subsequently reported new and continuing dizziness and eye discomfort, without headache or tinnitus. Brain MRI 3 months after starting ipi/nivo showed new sub-centimeter supra- and infratentorial enhancing and hemorrhagic lesions (Fig. 2a, b) despite significant improvement in systemic disease on concurrent chest and abdominal CT imaging (Fig. 2c). She also developed progressive vision loss and orbital pain which ultimately was not responsive to topical or intraocular steroid therapy. A final dose of nivolumab (240 mg) was then administered and she was continued on steroid therapy. The patient did not receive brain radiotherapy at any point during her treatment. As serial MR imaging one month later revealed only a solitary focus of enhancement (Fig. 2d), her persistent visual symptoms were felt to be on the basis of an immunotherapy-related adverse event rather than direct effect from intracranial metastatic disease. Subsequent evaluation by Ophthalmology revealed both uveitis and exudative retinal detachments suggestive of Vogt–Koyanagi–Harada disease. She was tapered off steroids without any significant change in her clinical exam or radiological findings at 5- month follow-up imaging (Fig. 2e).

## Discussion and conclusions

Melanoma metastases to the brain remain a significant clinical challenge for oncologists given their frequency and poor response to traditional therapy. Treatment has historically involved surgery and/or radiation, with typically poor response to cytotoxic chemotherapy. Before the availability of BRAF/MEK inhibitors and anti-CTLA-4/PD-1 drugs, median overall survival (OS) for metastatic melanoma was about 6 months, with 25% patients alive at 1 year [13]. Several phase II trials designed specifically for patients with metastatic melanoma and brain metastases [9–12, 14, 15] demonstrated that vemurafenib, dabrafenib, ipilimumab, nivolumab and pembrolizumab have intracranial clinical activity and may achieve improved survival in patients with brain metastases compared to benchmark survival rates in patients with metastatic melanoma with or without brain metastases [16]. Currently two ongoing phase II studies, the CheckMate 204 trial [14] and the anti-PD1 Brain Collaboration (ABC) trial [15], lend support to combined use of combined nivolumab and ipilimumab treatment in patients with metastatic melanoma with brain metastases, with objective intracranial response rates ranging from 42 to 55% and a 6 month progression free survival rate of 54–65%. While these and other trials suggest an expanding role for immunotherapeutics in metastatic melanoma, no study with survival as primary endpoint has been conducted so far with an adequate representation of patients with brain metastases.

Even less is known about the brain specific therapeutic responses and adverse events in this patient cohort. A recent meta-analysis in 2016 summarized findings from 24 phase II and III trials inclusive of metastatic melanoma patients with brain metastases, with clinical outcomes ranging from combined intra- and extracranial disease response, intracranial disease progression with extracranial response and dual progression [17]. Similarly, the development of intracranial hemorrhagic disease was also observed, although it is unclear if this represents the natural course of metastatic melanoma versus direct therapeutic effect.

Here we discuss two independent cases of intracranial metastases presenting after initiation of ipi/nivo in the setting of positive systemic disease. Although our case series only includes two patients, mixed disease progression is not unusual in metastatic melanoma and these cases raise the question of whether the initial immune activation and modulation of the blood brain barrier by ipi/nivo somehow “unmasks” previously clinically silent metastatic disease, akin to pseudoprogression on MRI, rather than new or progressive metastatic disease. The concept of pseudoprogression in response to CTLA-4/PD-1 inhibitors has been well described in systems outside the brain [18] however no intracranial correlate has yet to be fully documented.

Similarly, intralesional hemorrhage was observed in both patients while on ipi/nivo. While metastatic melanoma has a known propensity for spontaneous hemorrhage, it remains unclear if the rate of metastatic hemorrhage is increased under treatment of ipi/nivo, or if gross hemorrhage may reflect a brain specific adverse event. If the later proves true, implementation of a screening brain MRI prior to initiating immunotherapy could potentially help clinicians predict the onset and severity of intracranial adverse events but is unlikely to alter clinical management in the setting of treatable systemic disease and thus may be better reserved for investigation on a symptomatic basis. Regardless, these findings highlight the need for future work elucidating the specific therapeutic response of brain metastases to BRAF/MEK inhibitors and anti-CTLA/PD1 drugs, including delineation of the time course of disease response, the ideal therapeutic target windows in terms of the size and number of intracranial metastases, as well as the documentation of brain-specific adverse events. Additionally, while stereotactic radiosurgery (SRS) has been the standard of care to treat new brain melanoma metastases due to its efficacy in providing local control [19], the treatment paradigm of SRS first may warrant re-evaluation to avoid unnecessary delay of systemic immunotherapy related to corticosteroid use for treatment-related vasogenic edema.

## Abbreviations

CT: Computed tomography
CTLA 4: Cytotoxic T-lymphocyte-associated protein 4
Ipi: Ipilimumab
MRI: Magnetic resonance imaging
Nivo: Nivolumab
PD-1: Programmed death 1
SRS: Stereotactic radiosurgery
